# Protonation enhancement by dichloromethane doping in low-pressure photoionization

**DOI:** 10.1038/srep36820

**Published:** 2016-12-01

**Authors:** Jinian Shu, Yao Zou, Ce Xu, Zhen Li, Wanqi Sun, Bo Yang, Haixu Zhang, Peng Zhang, Pengkun Ma

**Affiliations:** 1 State Key Joint Laboratory of Environment Simulation and Pollution Control, Research Center for Eco-Environmental Sciences, Chinese Academy of Sciences, Beijing 100085, China; 2 University of Chinese Academy of Sciences, Beijing, China; 3 Shanghai Masteck Environment Co., Ltd, Shanghai, China

## Abstract

Doping has been used to enhance the ionization efficiency of analytes in atmospheric pressure photoionization, which is based on charge exchange. Compounds with excellent ionization efficiencies are usually chosen as dopants. In this paper, we report a new phenomenon observed in low-pressure photoionization: Protonation enhancement by dichloromethane (CH_2_Cl_2_) doping. CH_2_Cl_2_ is not a common dopant due to its high ionization energy (11.33 eV). The low-pressure photoionization source was built using a krypton VUV lamp that emits photons with energies of 10.0 and 10.6 eV and was operated at ~500–1000 Pa. Protonation of water, methanol, ethanol, and acetaldehyde was respectively enhanced by 481.7 ± 122.4, 197.8 ± 18.8, 87.3 ± 7.8, and 93.5 ± 35.5 times after doping 291 ppmv CH_2_Cl_2_, meanwhile CH_2_Cl_2_ almost does not generate noticeable ions itself. This phenomenon has not been documented in the literature. A new protonation process involving in ion-pair and H-bond formations was proposed to expound the phenomenon. The observed phenomenon opens a new prospect for the improvement of the detection efficiency of VUV photoionization.

Photoionization (PI), a widely used soft ionization technique, is usually coupled to various mass spectrometers for analyzing the chemical composition of samples^1^,^2^,^3^,^4^,^5^. Atmospheric pressure photoionization (APPI) is a new and highly attractive ionization technique^6^,^7^, which was developed ~10 years ago with the aim of improving the performance of liquid chromatography-mass spectrometry (LC-MS) for less polar compounds such as polycyclic aromatic hydrocarbons (PAHs). A krypton lamp, which emits VUV photons with energies of 10.0 and 10.6 eV, is usually chosen as the light source in APPI as it is cheap, compact, and robust. Different from classic vacuum photoionization, APPI shows characteristics more similar to those of chemical ionization (CI). The ionization mechanisms commonly observed in CI are also observed in APPI, such as the proton transfer reaction (PTR) and charge exchange. PTR typically takes place when the analyte in question has a higher proton affinity (PA), whereas charge exchange requires that the analyte possesses low ionization energy (IE).

Low-pressure photoionization (LPPI), defined as photoionization running under hundreds to thousands of Pa, has not been used as widely as APPI and conventional vacuum PI. LPPI has characteristics of both vacuum PI and APPI^8^. Apart from molecular ions, protonated ions were found to be dominant for polar compounds. The proton transfer reactions in LPPI can be expressed as follows:


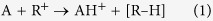



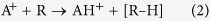


where A represents the analyte molecules and R is the reagent which offers a proton or hydrogen atom. The reagent could be the analyte or solvent molecules.

The use of dopants has been found to be very effective for enhancing the ionization efficiency of analytes^6^,^7^,^9^,^10^,^11^ in APPI and LPPI^12^ via charge exchange:


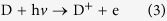



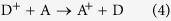


where D and A represent dopant and analyte molecules, respectively. Benzene (IE = 9.24 eV)^13^,^14^, acetone (IE = 9.70 eV)^10^,^15^,^16^,^17^, toluene (IE = 8.83 eV)^6^,^7^,^10^,^11^,^12^,^18^,^19^,^20^, and anisole (IE = 8.20 eV)^21^ are often employed as dopants due to their excellent photoionization efficiencies under illumination of the krypton lamp. The resulting analyte ions may subsequently react with other molecules via proton transfer. The detection sensitivity could be enhanced by ~100 times via doping^22^. However, these dopants cannot be applied to the detection of methanol (CH_3_OH, IE = 10.84 eV), ethanol (C_2_H_5_OH, IE = 10.48 eV), and acetaldehyde (C_2_H_4_O, IE = 10.23 eV) due to their higher IEs. Dichloromethane has been chosen as a dopant for characterizing the molecular structures of analytes via secondary ion–molecule reactions, rather than for enhancing ionization efficiency^23^.

Our previous studies revealed that LPPI with a specially designed photoionizer was super sensitive (~1000 counts/ppbv) to many organic compounds^24^,^25^,^26^. However, the LPPI detection efficiency for CH_3_OH, C_2_H_5_OH, and C_2_H_4_O is very low due to their low ionization efficiencies. In this paper, we report a new phenomenon: The detection efficiencies of the three small volatile organic compounds (VOCs) can be remarkably enhanced via CH_2_Cl_2_ doping. The results and experimental method are described in the following sections.

## Results

### Protonation enhancement of water and LPPI mass spectrum of CH_2_Cl_2_

**Figure 1:**
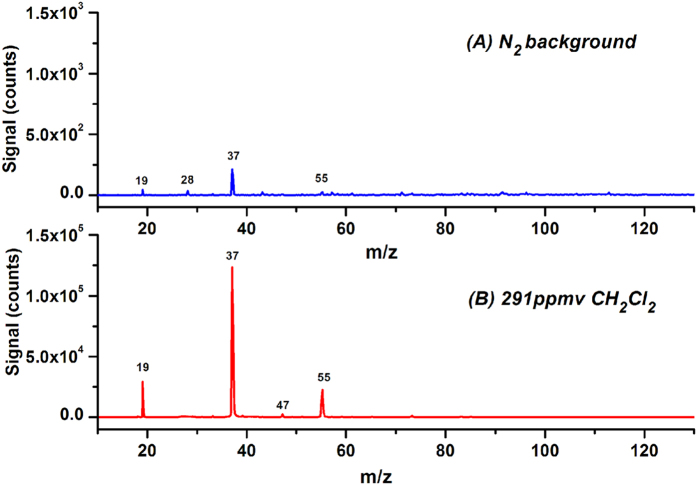
LPPI mass spectra of N_2_ before (**A**) and after (**B**) doping with 291 ppmv CH_2_Cl_2_.

Water (H_2_O) is an important protonation agent for PTR mass spectrometry. The IE of water is 12.62 eV, which indicates that it cannot be photoionized directly by the photons emitted from the krypton lamp. However, H_3_O^+^ (m/z 19, 45 counts), (H_2_O)_2_H^+^ (m/z 37, 214 counts), and (H_2_O)_3_H^+^ (m/z 55, 24 counts) were observed in the LPPI mass spectrum of N_2_, as shown in Fig. 1(A). The concentration of water in the test chamber was <5 ppmv, as a result of impurities in high-purity N_2_ gas. Protonation of acetonitrile (IE = 12.20 eV) was observed in APPI with a krypton lamp as the VUV light source by Marotta *et al*. The authors speculate that photon irradiation leads first to the isomerization of acetonitrile molecules, affording species that exhibit IEs <10 eV and that consequently are able to generate photoionization products^27^. The formation mechanism of protonated water and water clusters under illumination of 10.0 and 10.6 eV photons is not clear yet. In view of a tiny amount of N_2_^+^ (m/z 28, 34 counts) observed in Fig. 1(A), the photoelectrons in the photoionization region might lead to the formation of protonated water and water clusters. Figure 1(B) shows the mass spectrum obtained after injecting 291 ppmv CH_2_Cl_2_ into the chamber. Surprisingly, the signal intensities of H_3_O^+^, (H_2_O)_2_H^+^, and (H_2_O)_3_H^+^ increased to 2.92 × 10^4^, 1.24 × 10^5^ and 2.29 × 10^4^ counts, respectively. The signal intensity of protonated water was averagely enhanced by 481.7 ± 122.4 times, measured from three independent measurements. This phenomenon has never been reported.

CH_2_Cl_2_ is a common solvent used in organic analysis. The IE of CH_2_Cl_2_ is 11.33 eV. It cannot be directly ionized by the VUV photons emitted from the krypton lamp. As shown in Fig. 1(B), no noticeable ions were produced from direct photoionization of CH_2_Cl_2_. A small mass peaks at m/z 47 is assigned to ethanol residual in the test chamber or minor impurity in the CH_2_Cl_2_ reagent.

### Protonation enhancement of methanol, ethanol, and acetaldehyde

**Figure 2:**
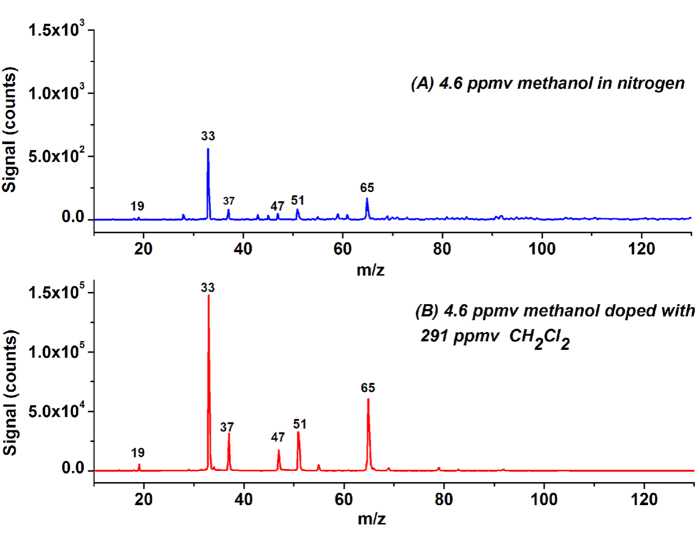
LPPI mass spectra of 4.6 ppmv methanol before (**A**) and after (**B**) doping with 291 ppmv CH_2_Cl_2_.

Methanol (CH_3_OH) is the simplest alcohol. Its IE is 10.84 eV, higher than the energy of the photons emitted from the krypton lamp. A weak signal of protonated methanol was observed when 4.6 ppmv methanol was sampled. Figure 2(A) shows the obtained LPPI mass spectrum of 4.6 ppmv methanol in nitrogen. The mass peaks at m/z 19, 37, and 55 correspond to H_3_O^+^, (H_2_O)_2_H^+^, and (H_2_O)_3_ H^+^, respectively. The mass peaks at m/z 33, 51 and 65 are assigned to (CH_3_OH)H^+^, (CH_3_OH·H_2_O)H^+^ and (CH_3_OH)_2_H^+^, respectively. The moderate mass peak at m/z 47 is assigned to ethanol, the impurity in the methanol reagent. The peak intensities of (CH_3_OH)H^+^ and (CH_3_OH)_2_H^+^ are 559 and 171 counts, respectively. It is reported in the literature that dimers of methanol (CH_3_OH)_2_ with IE equal to 9.72 eV coexist with methanol monomers under ambient conditions and that protonated methanol is generated from the dissociation of (CH_3_OH)_2_^+^^28^,^29^. Figure 2(B) shows the LPPI mass spectrum of 4.6 ppmv methanol doped with 291 ppmv CH_2_Cl_2_. The signal intensities of the mass peaks at m/z 33 and 65 reach 1.48 × 10^5^ and 6.06 × 10^4^ counts, respectively. The signal intensity of protonated methanol was averagely enhanced by 197.8 ± 18.8 times, measured from three independent measurements.

**Figure 3:**
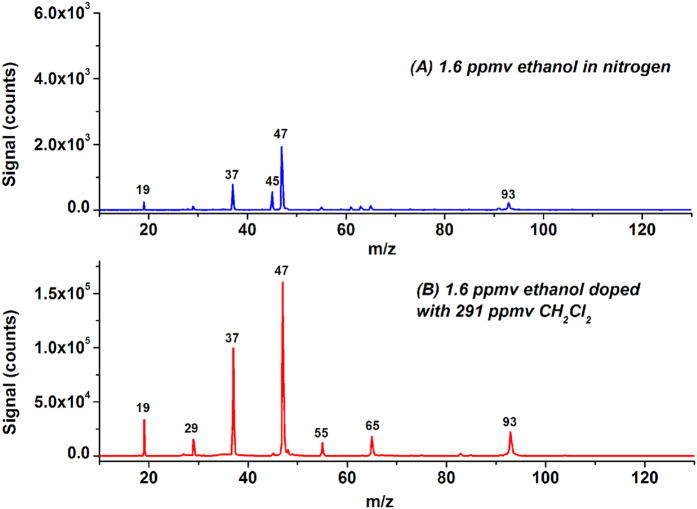
LPPI mass spectra of 1.6 ppmv ethanol before (**A**) and after (**B**) doping with 291 ppmv CH_2_Cl_2_.

The IE of ethanol (C_2_H_5_OH) is 10.48 eV, meaning it can be photoionized by the photons emitted from the krypton lamp (10.6 eV, 20%). Figure 3(A) shows the LPPI mass spectrum of 1.6 ppmv ethanol in nitrogen. As well as ions resulting from water and water clusters, mass peaks at m/z 45, 47, and 93 are assigned to ions produced from ethanol, i.e. C_2_H_5_O^+^ (551 counts), (C_2_H_5_OH)H^+^ (1923 counts), and (C_2_H_5_OH)_2_H^+^ (222 counts). The mass peak of protonated ethanol was the strongest peak. After doping with 291 ppmv CH_2_Cl_2_, the intensities of the mass peaks at m/z 47 and 93 shown in Fig. 3(B) increased to 1.61 × 10^5^ and 2.21 × 10^4^ counts, respectively. The signal intensity of protonated ethanol was averagely enhanced by 87.3 ± 7.8 times, measured from three independent measurements. The mass peak at m/z 45 slightly increased to 2765 counts, while the mass peaks at m/z 29 (1.54 × 10^4^ counts) and 65 (1.80 × 10^4^ counts) assigned to C_2_H_5_^+^ and (C_2_H_5_OH·H_2_O)H^+^ appeared.

**Figure 4:**
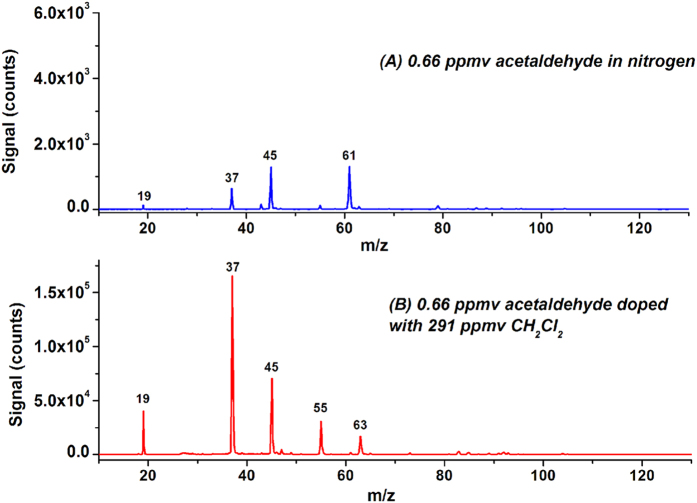
LPPI mass spectra of 0.66 ppmv acetaldehyde before (**A**) and after (**B**) doping with 291 ppmv CH_2_Cl_2_.

Acetaldehyde (C_2_H_4_O) is one of the most important aldehydes; it occurs widely in nature and is produced industrially on a large scale. The IE of acetaldehyde is 10.23 eV. Figure 4(A) shows the LPPI mass spectrum of 0.66 ppmv acetaldehyde in pure nitrogen. The mass peaks at m/z 45 and 61 are assigned to (C_2_H_4_O)H^+^ (1290 counts), and (C_2_H_3_O·H_2_O)^+^ (1307 counts), respectively. The molecular ion of acetaldehyde was not observed. Protonated acetaldehyde was the dominant ion. Figure 4(B) shows the LPPI mass spectrum of 0.66 ppmv acetaldehyde in nitrogen doped with 291 ppmv CH_2_Cl_2_. The signal intensity of protonated acetaldehyde (m/z 45) increased to 7.04 × 10^4^ counts, while the signal at m/z 61 slightly increased to 2107 counts. The signal intensity of protonated acetaldehyde was averagely enhanced by 93.5 ± 35.5 times, measured from three independent measurements. Additionally, a mass peak at m/z 63 assigned to (C_2_H_4_O·H_2_O)H^+^ (1.71 × 10^4^ counts) appeared.

**Figure 5:**
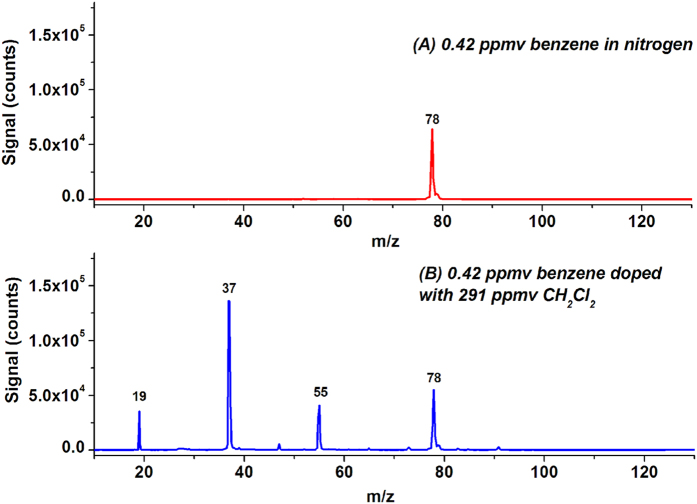
LPPI mass spectra of 0.42 ppmv benzene before (**A**) and after (**B**) doping with 291 ppmv CH_2_Cl_2_.

Benzene (C_6_H_6_) is an important chemical and atmospheric pollutant. Its IE is 9.24 eV, lower than the energy of VUV photons emitted from the krypton lamp. Benzene and its derivatives have excellent photoionization efficiencies under illumination of a krypton VUV lamp. Figure 5(A) shows the LPPI mass spectrum of 0.42 ppmv benzene. The mass peak at m/z 78 is assigned to ^12^C_6_H_6_^+^ (6.42 × 10^4^). Figure 5(B) shows the LPPI mass spectrum of 0.42 ppmv benzene in nitrogen doped with 291 ppmv CH_2_Cl_2_. The intensities of the mass peak at m/z 78 decreased by ~14% to 5.54 × 10^4^ counts. The fluctuation of the signal intensities at m/z 78 was observed in separate experiments. No signal enhancement at m/z 79 (protonated benzene) was observed in all experiments.

## Discussion

Pure CH_2_Cl_2_ in LPPI almost does not generate noticeable ions as shown in Fig. 1, which implies that the observed protonation enhancement is not attributed to charge exchange. In order to reveal the mechanism of protonation enhancement, the doping effects of H_2_, CH_4_, CHCl_3_, and CCl_4_ on the signals of methanol, ethanol, and acetaldehyde were also investigated. Among the four dopants, only CHCl_3_ yielded a weaker enhancement on protonation of methanol, ethanol, and acetaldehyde compared with CH_2_Cl_2_. Under illumination of the krypton lamp, CH_4_, CHCl_3_, CH_2_Cl_2_, and CCl_4_ have relatively strong absorption (~10^−17^ cm^2^) and are excited to Rydberg states^30^,^31^, while H_2_ does not have absorption^32^. Shaw *et al*. reported that ion-pair states were observed in halogenated methanes excited by VUV light and ion pair states even existed below ionization potentials^33^. We speculate that CHCl_3_ and CH_2_Cl_2_, excited by the krypton lamp, may form the ion-pair states of [H^+^−CCl_3_^−^] and [H^+^−CHCl_2_^−^], which facilitate protonation. Other dopants including H_2_, CH_4_, and CCl_4_ do not meet the combined conditions of formation of ion-pair states and existence of H atoms.

Table 1 lists IEs, PAs, molecular dipole moments, H-bond formation possibilities, and protonation enhancements of the compounds investigated. It is very interesting that protonation of benzene and self-protonation of dichloromethane were not observed in the experiment, while water and other three organics had significant protonation enhancements. The difference observed in protonation enhancements cannot be addressed simply by proton affinities or molecular dipole moments of the compounds. It is enlightening that the observed protonation enhancements of the compounds are coincident with their abilities to form a H bond as a H acceptor as shown in Table 1. The four compounds, water, methanol, ethanol, and acetaldehyde, are all capable of forming a H bond as a H acceptor, while benzene and dichloromethane are not. These phenomena may imply that the compounds are not protonated by free protons or protonated molecules. Based on experimental observations and the analyses above, we speculate that the following process might take place during CH_2_Cl_2_ doping:


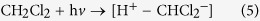



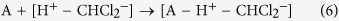



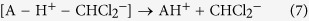


where [H^+^−CHCl_2_^−^] represents an ion-pair state, [A−H^+^−CHCl_2_^−^] sketches a complex formed via a H bond, and A denotes analyte molecules, i.e. molecules of water, methanol, ethanol, and acetaldehyde. The proposed scenario of protonation enhancement is as follows: 1. CH_2_Cl_2_ excited by VUV light transforms into an ion-pair state ([H^+^−CHCl_2_^−^]); 2. The analyte molecule collides with [H^+^−CHCl_2_^−^] and forms a complex [A−H^+^−CHCl_2_^−^] via a H bond; 3. The detachment of the proton from CH_2_Cl_2_ leads to the formation of a protonated analyte molecule (AH^+^) and CHCl_2_^−^. This hypothesis rationalizes all the experimental observations. To the best of our knowledge, protonation via collision with excited-state molecules has not yet been documented. The heat of reaction (Δ_r_H°) of deprotonation of CH_2_Cl_2_ (CH_2_Cl_2_ = CHCl_2_^−^ + H^+^) is ~16.3 eV^34^. Considering the photon energy of VUV light (~10 eV) and PAs of analyte molecules (in the range of 7–9 eV)^35^, the total process of Reactions 5 to 7 is exothermic for most VOCs. Though the authenticity and intrinsic mechanism of the process still needs further elaborate investigation, the observed phenomenon opens a new prospect for the improvement of the detection efficiency of VUV photoionization.

## Methods

The experimental setup has been described in detail elsewhere^25^. Briefly, it mainly consisted of a 120 L test chamber and a LPPI mass spectrometer.

The 120 L test chamber was mainly built with an open-head stainless steel drum and covered with a thin Tedlar bag to ensure one atmospheric pressure during sampling. A stainless steel fan driven by a magnetic field was placed at the bottom of the test chamber to ensure quick mixing. Nitrogen was used as the buffer gas. An oil-free pump was used as the drain pump. Two mass flow controllers were used for gas samples. All experiments were performed under ambient atmospheric pressure and room temperature.

The LPPI mass spectrometer was recently developed in our laboratory. It characterizes with a LPPI source with an optical baffle and a short reflectron time-of-flight mass spectrometer. The body of the LPPI source was a cylindrical stainless steel cavity 6 mm in diameter and 35 mm in length. A radio frequency-driven krypton VUV lamp was used as the VUV light source and coupled to the cylindrical stainless steel cavity with an MgF_2_ window. The optical baffle was placed at the exit of the photoionization source to prevent the VUV light entering the mass spectrometer. The LPPI source was passivated with ~600 ppm CH_2_Br_2_ under illumination of VUV light for ~8 hours to suppress photoelectron formation in the experiment. The krypton lamp was laboratory-assembled and emitted VUV photons with energies of 10.0 eV (~80%) and 10.6 eV (~20%). The sample gas was introduced into the photoionization source and controlled by a needle valve. The sample flow was maintained at ~1 cm^3^ s^−1^. The pressure in the photoionization source was 500–1000 Pa. The mass spectrometer was a simple V-shaped time-of-flight mass spectrometer with a free-field flight distance of 460 mm. The cycle time of detection was 10 s.

In the experiments, a small amount of bottle-contained chemical was first injected into the test chamber. Then, 100 μL CH_2_Cl_2_ was injected into the test chamber and the mass spectra were subsequently acquired after each injection. The amount of methanol, ethanol, acetaldehyde, and benzene injected into the nitrogen-filled test chamber was 1.0, 0.5, 0.5, and 0.2 μL, respectively. The resulting mixing ratios for the prepared gases were 4.6, 1.6, 0.66, and 0.42 ppmv, respectively. The injection of 100 μL CH_2_Cl_2_ resulted in 291 ppmv in the mixing ratio.

In this study, methanol (A. R., Sinopharm), ethanol (A. R., Sinopharm), acetaldehyde (40% in water, Sinopharm), benzene (A. R., Beijing Shiji), CH_2_Cl_2_ (HPLC grade, Cleman Chemical), CHCl_3_ (A. R., Beijing Shiji), and CCl_4_ (A. R., Sinopharm) were used. High-purity nitrogen (>99.99%), hydrogen (>99.999%), and methane (>99.9%) were purchased from Beijing Huayuan Gas Co., Ltd.

## Additional Information

**How to cite this article**: Shu, J. *et al*. Protonation enhancement by dichloromethane doping in low-pressure photoionization. *Sci. Rep.* **6**, 36820; doi: 10.1038/srep36820 (2016).

**Publisher's note:** Springer Nature remains neutral with regard to jurisdictional claims in published maps and institutional affiliations.

## Figures and Tables

**Table 1:** Ionization energies (IEs), proton affinities (PAs), molecular dipole moments, H-bond formation possibilities, and protonation enhancements of the compounds investigated.

Compounds	Ionization Energy^a^ (eV)	Proton Affinity^a^ (kJ/mol/eV)	Molecular Dipole Moment^b^ (10^−30^ C·m)	H-bond Formation Possibilities as H Acceptor	Protonation Enhancement by CH_2_Cl_2_^c^ (times)
H_2_O	12.62	691/7.22	6.2	Yes	481.7±122.4
CH_3_OH	10.84	754.3/7.82	5.5	Yes	197.8±18.8
C_2_H_5_OH	10.48	776.4/8.05	5.7	Yes	87.3±7.8
C_2_H_4_O	10.23	768.5/7.97	8.3	Yes	93.5±35.5
C_6_H_6_	9.24	750.4/7.78	0	No	0
CH_2_Cl_2_	11.33	628/6.51	6.0	No	0

^a^http://webbook.nist.gov/.

^b^http://www.kayelaby.npl.co.uk/.

^c^Obtained from three independent measurements.
